# A Prototype Patient-Maintained Propofol Sedation System Using Target Controlled Infusion for Primary Lower-Limb Arthroplasty

**DOI:** 10.1007/s10916-019-1377-3

**Published:** 2019-06-26

**Authors:** James Sprinks, Frank Worcester, Philip Breedon, Paul Watts, David Hewson, Nigel Bedforth

**Affiliations:** 1 Medical Engineering Design Research Group, Nottingham Trent University, Nottingham, UK; 2 Department of Anaesthesia and Critical Care, Nottingham University Hospitals NHS Trust, Nottingham, UK

**Keywords:** Propofol sedation, Patient-maintained sedation, Target controlled infusion (TCI), Regional Anaesthesia, Joint arthroplasty

## Abstract

Each year, many operations in the UK are performed with the patient awake, without the use of general anaesthesia. These include joint replacement procedures, and in order to reduce patient anxiety, the supervising anaesthetist delivers the sedative propofol intravenously using a target-controlled infusion (TCI) device. However, it is clinically challenging to judge the required effect-site concentration of sedative for an individual patient, resulting in patient care issues related to over or under-sedation. To improve the process, patient-maintained propofol sedation (PMPS), where the patient can request an increase in concentration through a hand-held button, has been considered as an alternative. However, due to the proprietary nature of modern TCI pumps, the majority of PMPS research has been conducted using prototypes in research studies. In this work, a PMPS system is presented that effectively converts a standard infusion pump into a TCI device using a laptop with TCI software. Functionally, the system delivers sedation analogous to a modern TCI pump, with the differences in propofol consumption and dosage within the tolerance of clinically approved devices. Therefore, the Medicines and Healthcare products Regulatory Agency (MHRA) has approved the system as a safe alternative to anaesthetist-controlled TCI procedures. It represents a step forward in the consideration of PMPS as a sedation method as viable alternative, allowing further assessment in clinical trials.

## Background

A considerable number of operations (over 800,000) performed annually in the UK are conducted in the presence of an anaesthetist, but without using general anaesthesia [1]. Lower limb surgical arthroplasty largely contributes to this number, with the most common procedures being hip and knee replacement (~200,000 such replacements were carried out in 2016, excluding Scotland [2]). In such cases, regional anaesthesia (numbing part of the body) using a spinal anaesthetic provides excellent operating conditions whilst reducing some of the risks associated with general anaesthesia [3]. However, whilst a minority of patients are accepting of experiencing surgery awake, a substantial number are not as keen and can experience varying levels of anxiety either before or during the operation [4, 5]. As well as the obvious negative impact on patient experience, procedural anxiety can be associated with negative surgical outcomes including post-operative pain [6]. To negate this issue, several techniques have been tried and found to be effective at reducing patient anxiety including visual distraction [7], patient education [8] and music therapy [9].

**Fig. 1:**
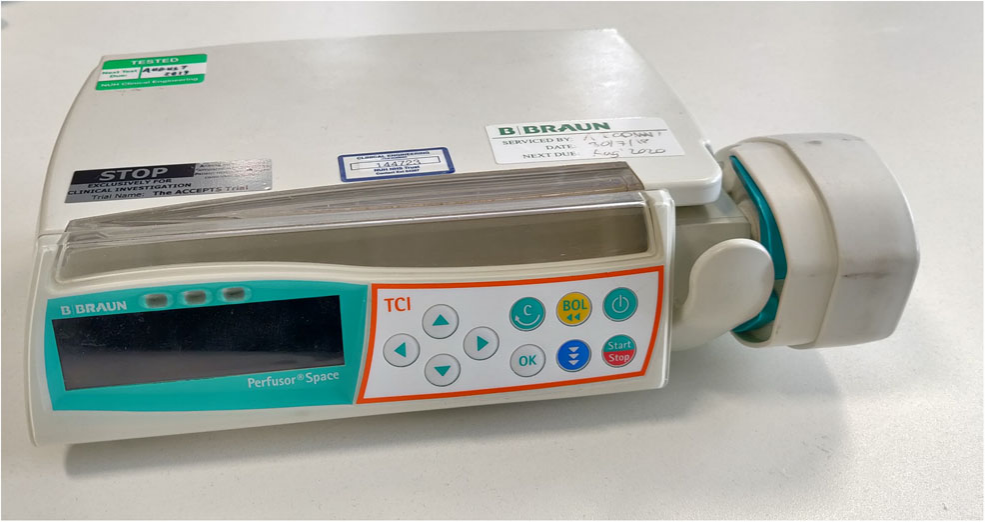
B.Braun Perfusor® Space Target Controlled Infusion Device

In order to reduce patient anxiety during lower-limb arthroplasty, anaesthetists commonly use intravenously delivered sedation. This is provided by the supervising anaesthetist, with one option being administering propofol using computer-assisted Target Controlled Infusion (TCI) [10]. Propofol is a short-acting general anaesthetic and sedative agent with a rapid onset of action of approximately 30 s. It was first authorised for clinical use in the UK in 1986 (FDA approval in 1989), renewed in 2004 [11]. Whilst midazolam is perhaps more commonly used, the advantages of propofol by TCI include more rapid patient wake-up, less post-procedural confusion and delirium, and the ability to easily convert to general anaesthesia if required [12]. In such a system, a target-controlled infusion device delivers an infusion rate of propofol that varies over time to achieve a specified plasma or effect-site (i.e. brain) drug concentration (Fig. 1).

However, risks do exist regarding customisable drug administration such as TCI. These include the incorrect input of patient demographics resulting in inaccurate drug dosing, or the incorrect selection of TCI model for the drug to be infused i.e. placing a syringe containing a different drug into a device programmed for propofol delivery, or vice versa [13]. Additionally, the required effect-site concentration for an individual patient can be difficult to determine, as anaesthetists can often misjudge the patients’ anxiety levels [14]. This could result in either patients feeling under-sedated, causing the patient to be unnecessarily anxious, or over-sedated; both of which can promote adverse physiological states [15].

In order to improve the sedation process, patient-maintained propofol sedation (PMPS) is an alternative mechanism to anaesthetist control. A TCI system is again utilised, but instead of being anaesthetist controlled, the effect-site concentration of sedation is manipulated by the patient using a handheld button. The use of PMPS or patient-controlled sedation has previously been investigated in a number of small case series, including dental [16], endoscopy [17] and outpatient surgical [18], showing positive results regarding sedation concentration, patient anxiety levels and recovery time. Our group has previously conducted a pilot study of this sedative technique [19]. However, to date there has been only a small number of randomised controlled trials of PMPS in a clinical setting [20–22], of which none have used modern propofol TCI pumps with the pharmacokinetic algorithm ‘built in’ as an option (i.e. Schnider effect-site modelling [10]) in comparing PMPS against the anaesthetist-controlled standard.

This is, in part, because modern TCI infusions devices (such as the B. Braun Space shown in Fig. 1) are normally locked down and proprietary in design, meaning there is little or no flexibility in terms of integrating with or amending software protocols and interfaces [23]. Due to this, and the fact that commercially produced TCI pumps do not incorporate a patient-triggered interface, PMPS research studies have used specifically developed prototypes. These include the use of an infusion device connected to a computer running research software [20, 22], or an infusion device incorporated with a TCI microprocessor and patient interface [21]. Whilst existing studies have reported the impact of PMPS in terms of clinical and patient outcomes, there has been very little technical explanation regarding the development, integration, testing and deployment of TCI enabled PMPS systems.

In this work, a PMPS system utilising a B. Braun Perfusor**®** fm infusion device [24] connected to a laptop running bespoke TCI software and interfacing with a patient-controlled button device is presented. The system allows a direct comparison for surgical procedures between PMPS and anaesthetist-controlled propofol sedation (ACPS), the key goal of the associated National Institute for Health Research (NIHR) project [25] for which it was designed. Firstly the system architecture is described, including the hardware, software and communication methods employed. Additionally, the design and development of the system interface is considered, in order to ensure patient safety and reduce risk; a prerequisite for gaining Medicines and Healthcare Products Regulatory Agency (MHRA) approval for clinical trial deployment in the UK [26]. Finally, the performance of the proposed PMPS system is compared to a modern TCI integrated infusion pump, in terms of propofol dosage and total consumption for different patient demographics and target effect-site levels.

## Methods

In this section the layout of the PMPS system, its hardware, and communication devices are described. The software developed in order to drive the sedation pump, incorporating the Schnider TCI algorithm to maintain a given effect-site sedation level is also presented. Finally the system interface design and its testing is considered, including key usability and safety functionality in order to gain MHRA approval for clinical use.

### PMPS system layout

**Fig. 2:**
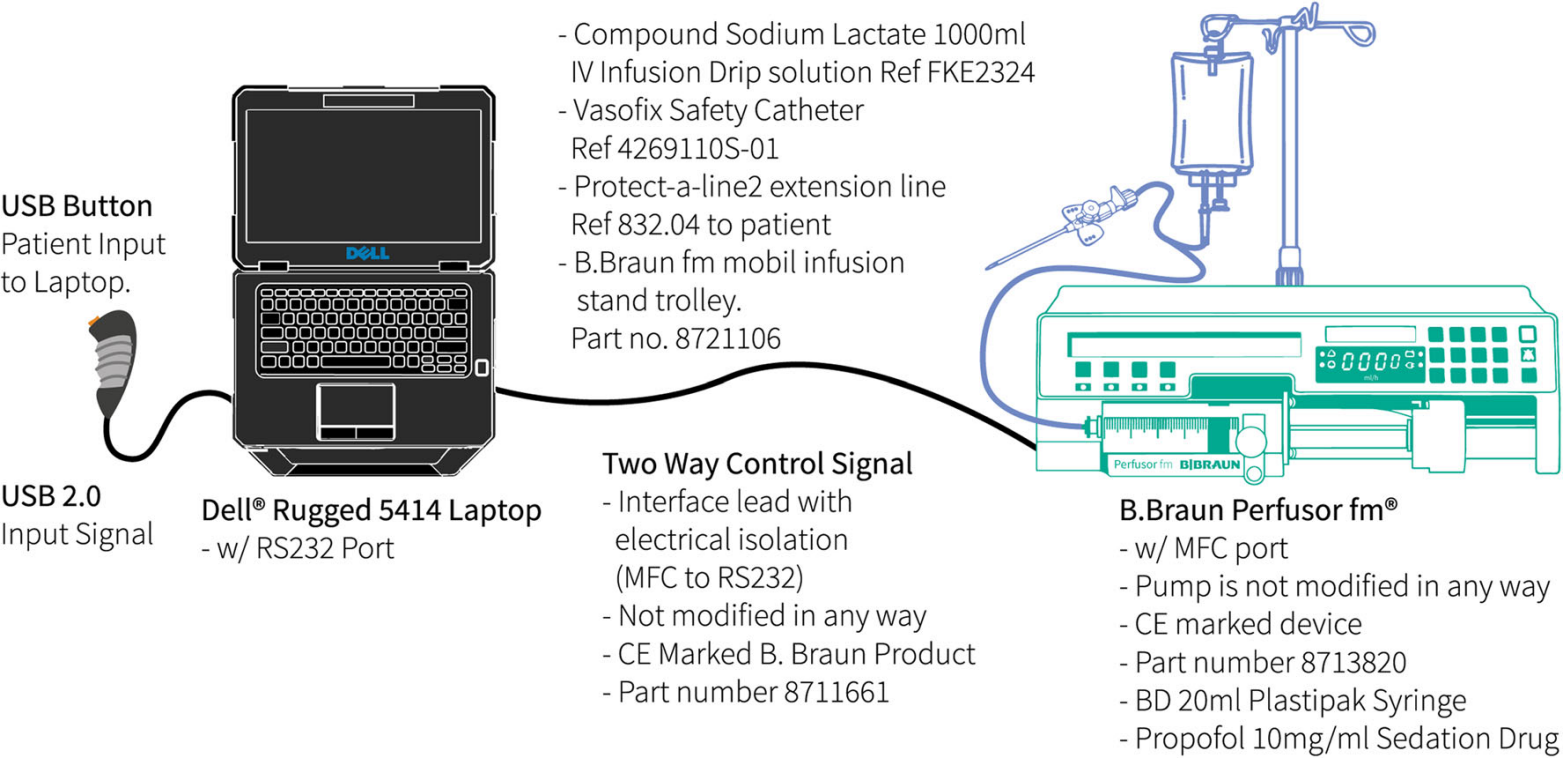
PMPS system layout and components

The layout of the PMPS system, including its hardware and communication methods is set out in Fig. 2. A laptop is connected to a B. Braun Perfusor**®** fm infusion device via a standard RS-232 serial port allowing two-way data transfer. The laptop contains software that implements the TCI algorithm, in order to calculate the required infusion rate for the target effect-site propofol concentration. It is modified to accept patient demand for an increase in sedation, via a USB connected button. In response to the laptop’s software-generated instructions, the pump delivers the appropriate infusion rate, with infusion data saved on the laptop in comma-separated values (csv) format, for data analysis.

### PMPS software design

The PMPS system’s software controls the sedation process, by responding to patient requests for increased sedation, calculating the required infusion rate in order to achieve it, and monitoring if it is safe to command the infusion pump to respond as appropriate. The software has been built as a standard Microsoft Windows application, ensuring it is intuitive to use for the supervising anaesthetist. In order to calculate the required infusion rate of propofol for a given concentration, current TCI systems are pre-programmed with either the Marsh [27] or Schnider [10] pharmacokinetic models. Whilst the two models differ in terms of how they calculate initial bolus and maintenance infusion rates, the Schnider model is preferred over the Marsh model for effect-site concentration targeting [28]. As such, it is the Schnider model that has been incorporated into the PMPS software, based on the mathematical equations and code of STANPUMP [29]. Fully open-source, STANPUMP is a computer program for driving an infusion pump, freely available to investigators and anaesthetists for research and clinical purposes.

**Fig. 3:**
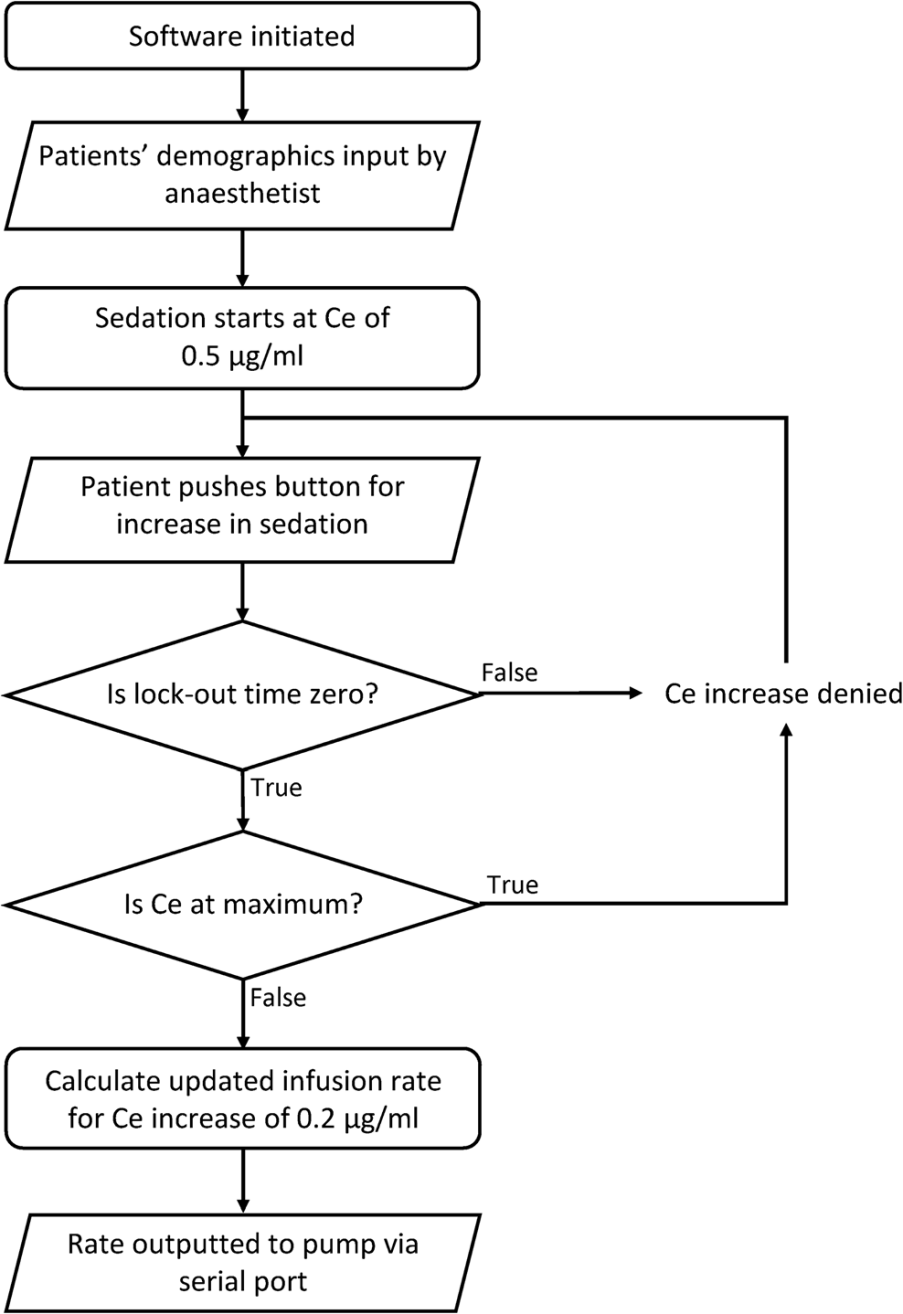
PMPS software flowchart showing response to patient request for sedation increase

The Schnider pharmacokinetic model requires patient weight, age, height, and lean body mass (LBM) (calculated from weight, gender and height) in order to calculate the infusion rate for a given effect-site concentration. Therefore, the first action the PMPS software undertakes is to request patient information to be inputted by the supervising anaesthetist. Once completed, and the anaesthetist and patient chose to begin sedation, the software starts sedation at an infusion rate in order to achieve a baseline effect-site concentration (Ce) of 0.5 μg/ml. A flowchart of how the PMPS software reacts to patient requests for increased sedation, the processes initiated and the decisions considered is shown in Fig. 3.

After initial setup and sedation commencement at a target Ce of 0.5 μg/ml, the PMPS software is then ready to respond to input from the patient button. When pressed by the patient (a single click), the software checks two conditions to ensure it is safe to increase the sedation level. Firstly, when the sedation starts, and every time sedation is increased through a patient button press, a lockout time of 2 min starts to count down. Within this lockout time, any button press requesting an increase in Ce is denied, to make sure the sedation level cannot be increased too quickly. Secondly, the software checks if the maximum Ce level has been reached. For the purpose of this project this has been set at 2.0 μg/ml, and if reached, again the PMPS software denies the request for an increase in Ce.

If both conditions are met to allow a sedation increase, the software uses the Schnider model to calculate the required infusion rate to maintain an increase of 0.2 μg/ml in target Ce level (e.g. 0.5 to 0.7 μg/ml, as extensively studied and deemed appropriate in previous research [30]). This data is then outputted to the B. Braun Perfusor**®** fm infusion pump through the serial port, and the pump responds accordingly. If the patient does not press the button to request an increase in sedation for 15 min, the target Ce level decrements by 0.1 μg/ml. This is to balance the need to maintain a satisfactory sedation level, avoiding the unpleasant sensation for a patient of going to sleep, then waking up, then going to sleep; with minimising their drug exposure over the course of the surgery to increase the speed of patient wake-up time after the operation is complete [31].

### PMPS anaesthetist interface

**Fig. 4:**
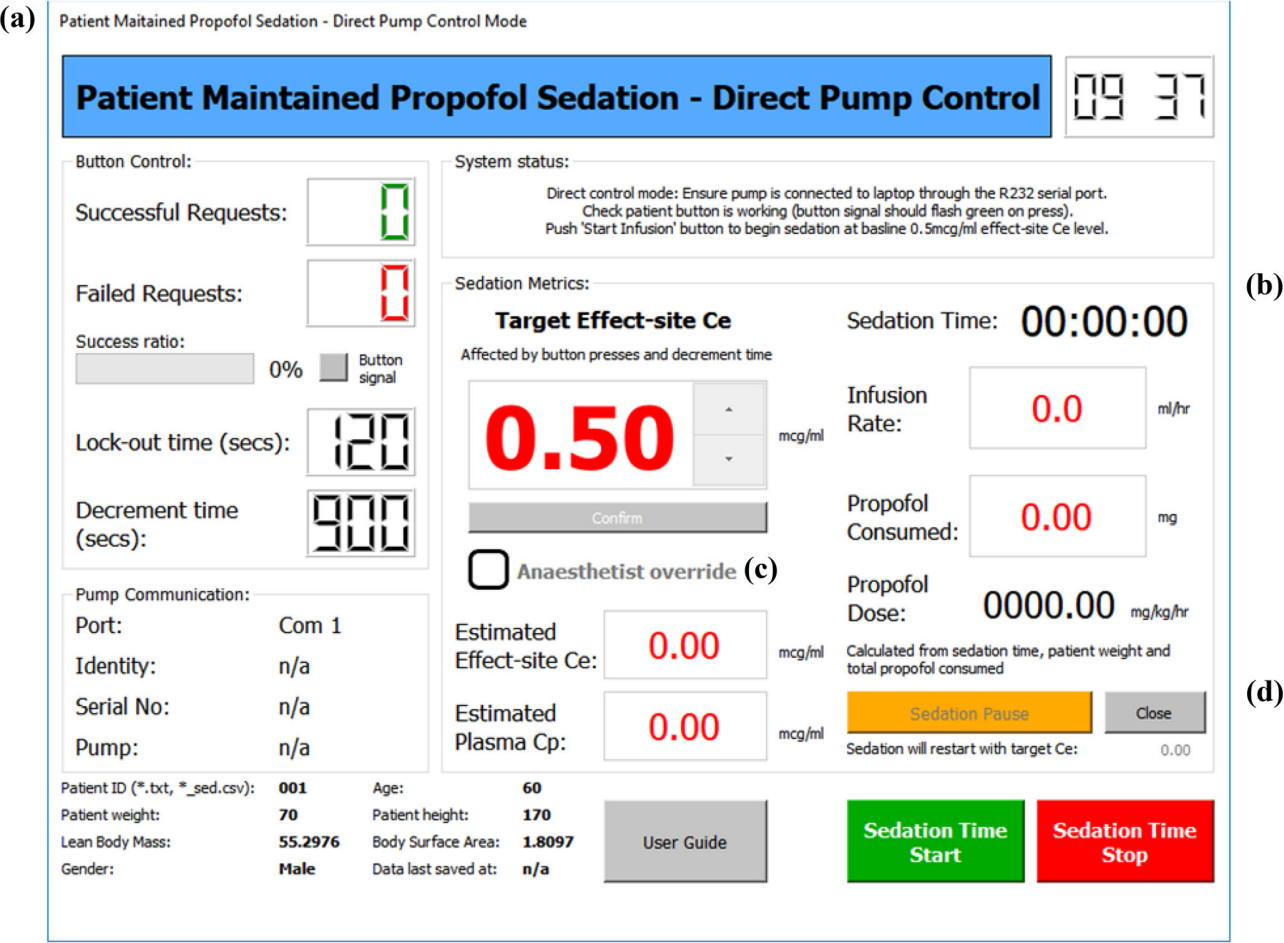
PMPS anaesthetist interface with the following displays: (**a**) – Button control display showing successful and denied patient request for increased sedation; (**b**) – Sedation metrics display showing sedation information; (**c**) – Anaesthetist override button allowing the anaesthetist to take system control; (**d**) – Sedation pause button allowing the anaesthetist to pause sedation

The PMPS system allows two-way communication between the software and the infusion pump, allowing data to be taken from the pump and displayed to the supervising anaesthetist through an interface on the system laptop (Fig. 2). The advantage of such an approach is that key sedation information can be presented on a single screen, without the need to navigate a number of sub-menus as with current commercially produced pump displays, therefore reducing the mental effort required for operation [32]. Additionally, information distinct to the PMPS process is also displayed. The PMPS anaesthetist interface shown in Fig. 4 has been developed in conjunction with practising anaesthetists [33], to ensure that the most relevant sedation information and functionality is displayed and included to ensure patient safety.

The PMPS interface consists of several sections that display different information pertinent to the patient-maintained sedation process. The button control display (see (a) in Fig. 4) informs the supervising anaesthetist of the number of sedation increase requests made by the patient, both successful and failed, and the ratio between the two so that the anxiety level of the patient can be broadly deduced. The sedation metrics display ((b) in Fig. 4) displays all of the key sedation metrics on screen simultaneously. An anaesthetist override button ((c) in Fig. 4) has been included on the PMPS interface to allow the anaesthetist to take complete control in the advent of a medical issue. On selecting this function, the patient button is de-activated, and the anaesthetist can raise and lower the target effect-site propofol concentration as they see fit. Finally, the sedation pause button ((d) in Fig. 4) allows the supervising anaesthetist to temporarily pause the sedation process (i.e. set the infusion rate to zero). This is so syringe changes or patient transfer from the anaesthesia room to the operating theatre can take place, and when completed the sedation will restart at the target effect-site concentration previously set before the pause.

## Results

In order to ensure the PMPS system delivers sedation correctly as per the Schnider TCI algorithm, the performance of the system has been compared to that of a commercially produced TCI pump (the B. Braun Perfusor**®** Space shown in Fig. 1). This has been done in terms of the amount of propofol consumed and the dosage rate during the sedation process for a number of patient metrics at differing target effect-site concentrations. In order to ensure the patient metrics and sedation levels are representative of primary lower-limb arthroplasty operations, the following simulations replicate sedation procedures that have taken place at the Nottingham University Hospitals NHS Trust. Table 1 shows the metrics of the patients used for the two sedation simulations. The lean body mass were calculated using the James formula [34], which is used for Schnider model TCI calculations [28].

**Table 1:** Patient metrics for the PMPS system test sedation simulations

Patient ID	Gender	Age (years)	Height (cm)	Weight (kg)	Lean Body Mass (kg)
A	Male	71	166	85	59.94
B	Female	68	161	65	45.43

**Fig. 5:**
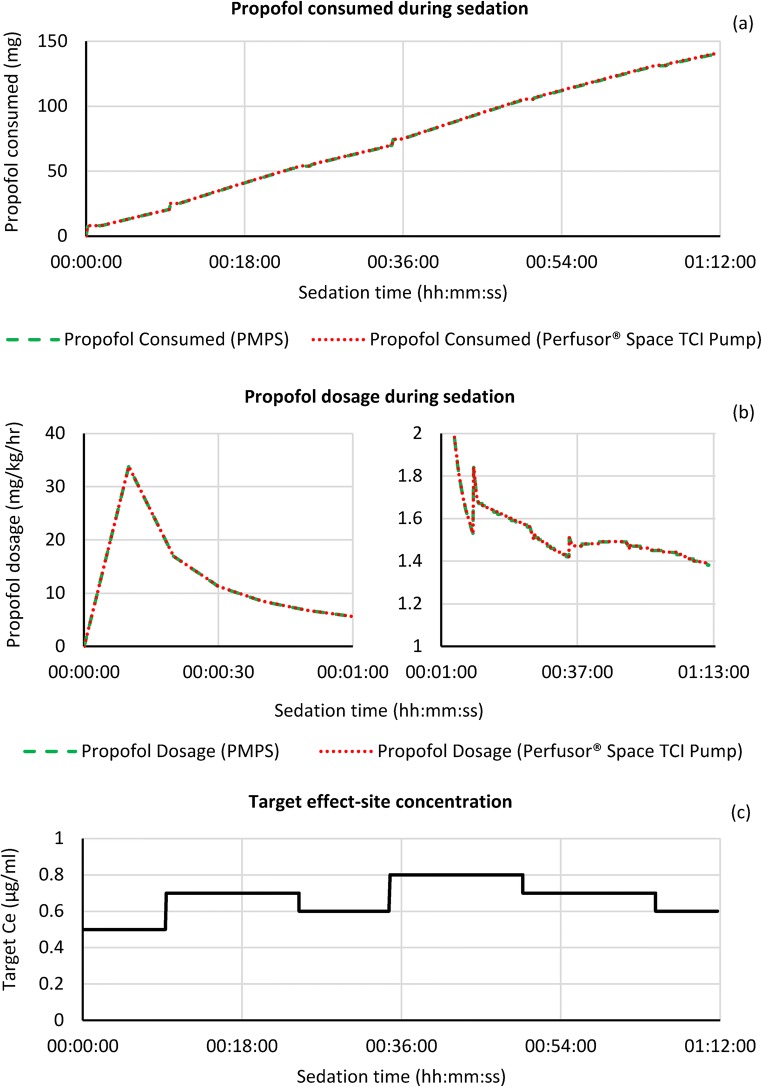
Propofol consumed (**a**), dosage – loading dose over 1st minute and target maintenance for remainder of sedation period (**b**) and target effect-site concentration (**c**) during the sedation process for simulation A

**Fig. 6:**
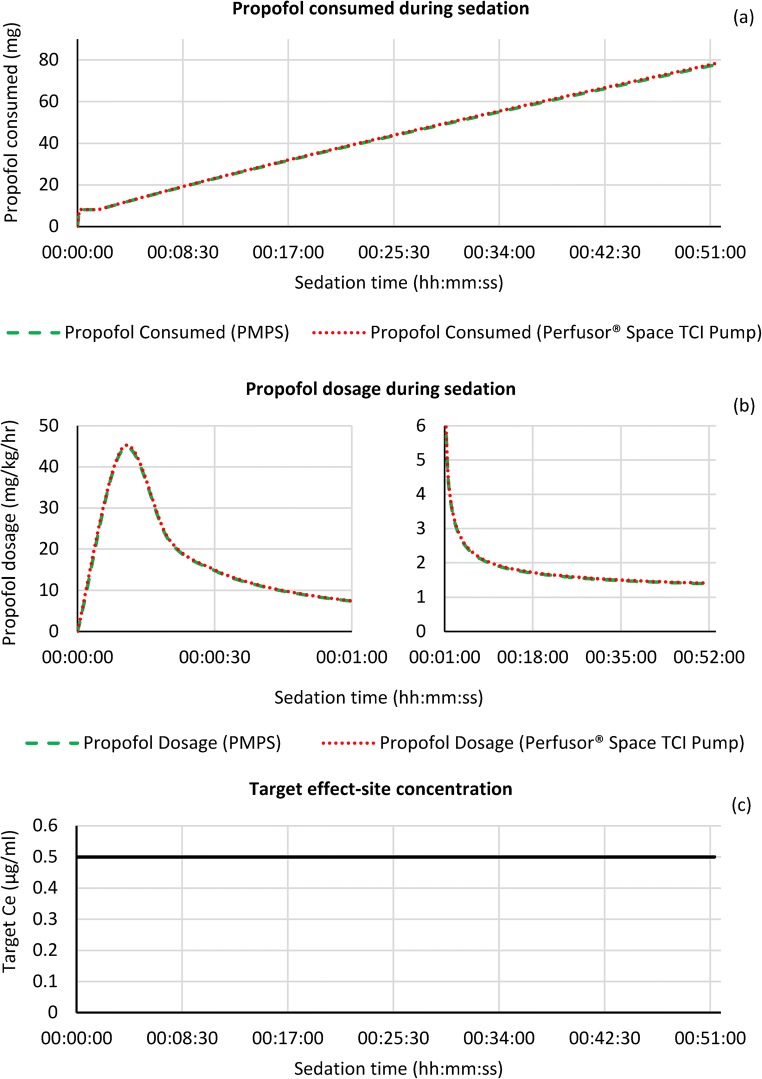
Propofol consumed (**a**), dosage – loading dose over 1st minute and target maintenance for remainder of sedation period (**b**) and target effect-site concentration (**c**) during the sedation process for simulation B

The following graphs shown in Figs. 5 and 6 show the sedation metrics (propofol consumed, propofol dosage and target effect-site concentration) for simulations A and B respectively. The propofol consumed is measured in milligrams (mg), whilst dosage is the simulated amount of propofol that would be consumed by the simulated patient per hour, normalised by their actual body weight (mg/kg/h).

During simulation A, the target Ce started at the base level of 0.5 μg/ml, increasing to 0.8 μg/ml through two button presses up to 36 min, with one decrement occurring at around the 20 min mark. No more increases were requested by the patient, with the target Ce decrementing twice to 0.6 μg/ml until the end of the operation 12 min later. As can be seen for both the amount of propofol consumed and the dosage, the profile for the PMPS system and the Perfusor**®** Space TCI pump is near identical. At the end of the sedation period the total amount of propofol consumed was 140.49 versus 140.91 mg for the PMPS system and the TCI pump respectively (a difference of 0.3%), whilst the final propofol dosage was 1.38 mg/kg/h for both.

Simulation B represents a calmer patient, who did not request an increase in sedation throughout the surgery. The target Ce started at the base level of 0.5 μg/ml, and remained at that level with no increase requests or decrements (as the target Ce was already at the lowest level) until the end of the operation. Again the profile of propofol consumed and dosage is near identical for both systems. At the end of the sedation period the total amount of propofol consumed was 77.77 versus 78.40 mg for the PMPS system and TCI pump respectively (a difference of 0.8%), whilst the final propofol dosage was 1.39 versus 1.41 mg/kg/h (a difference of 1.2%).

## Discussion

A PMPS system was created using a laptop running TCI software controlling a standard infusion pump to run as a modern TCI pump. This overcame the proprietary, locked-down designs of modern TCI pumps, and allowed the addition of a patient interface, allowing the comparison of PMPS and ACPS. The PMPS system delivers propofol sedation in a way analogous to a commercially available TCI pump when running simulated primary lower-limb arthroplasty procedures. In the simulations made over different patient metrics and target effect-site concentration profiles, the largest difference between the PMPS system and the Perfusor**®** Space TCI pump was 0.6 mg in terms of propofol consumed and 0.02 mg/kg/h in terms of dosage; a smaller variation than the tolerance of the commercial device [35]. Additionally, the inclusion of a laptop allowed an interface to be developed that includes all the data required by the supervising anaesthetist on a single display, reducing mental load and fatigue.

The PMPS system presented performed equivalently to a commercially produced TCI device in simulated sedation for lower-limb arthroplasty operations. The system will therefore allow comparison of PMPS and ACPS techniques in a medically approved, clinical trial setting.
